# Drivers Analysis of CO_2_ Emissions from the Perspective of Carbon Density: The Case of Shandong Province, China

**DOI:** 10.3390/ijerph15081762

**Published:** 2018-08-16

**Authors:** Feng Dong, Jingyun Li, Yue-Jun Zhang, Ying Wang

**Affiliations:** 1 School of Management, China University of Mining and Technology, Xuzhou 221116, China; 15852487068@163.com (J.L.); 15715201640@163.com (Y.W.); 2 Business School, Hunan University, Changsha 410082, China

**Keywords:** carbon density, LMDI, Kaya identity, structural change

## Abstract

Against the backgrounds of emission reduction targets promised by China, it is crucial to explore drivers of CO_2_ emissions comprehensively for policy making. In this study, Shandong Province in China is taken as an example to investigate drivers in carbon density by using an extended Kaya identity and a logarithmic mean Divisia index model (LMDI) with two layers. It is concluded that there are eight positive driving factors of carbon density during 2000–2015, including traffic congestion, land urbanization, etc., and seven negative driving factors comprising energy intensity, economic structure, etc. Among these factors, economic growth and energy intensity are the main positive and negative driving factor, respectively. The contribution rate of traffic congestion and land urbanization is gradually increasing. Meanwhile, 15 driving factors are divided into five categories. Economic effect and urbanization effect are the primary positive drivers. Contrarily, energy intensity effect, structural effect, and scale effect contribute negative effects to the changes in carbon density. In the four stages, the contribution of urbanization to carbon density is inverted U. Overall, the results and suggestions can give support to decision maker to draw up relevant government policy.

## 1. Introduction

Carbon (C) accounts for approximately 50% biomass. With the advancement of technology, on the one hand, carbon materials are widely used, such as numerous applications of amorphous carbon [1]. On the other hand, excessive emissions of carbon compounds (carbon dioxide emissions) cause many climate problems. As economic development accelerates, excessive fossil energy consumption caused by extended energy demand has led to a large amount of greenhouse gas emissions, making global warming become one of the main problems threatening human activities. In 2013, the Climate Change Performance Index (CCPI) evaluated and compared the performance of climate protection in 58 countries that contributed more than 90% of global CO_2_ emissions. In this assessment, Iran and Saudi Arabia ranked second to last and last, respectively [2]. The Intergovernmental Panel on Climate Change (IPCC) also has predicted that the global temperature will increase 1.4–5.8 °C during 1990–2100. This level of climate warming is a non-negligible problem. Meanwhile, recent deforestation in the tropics leaded to an annual atmospheric flux of 1.5 Pg C [3]. The climate problem cannot be ignored. On the one hand, the environment is being destroyed, on the other hand, greenhouse gases are excessively discharged. CO_2_ emissions as the main constitution of Greenhouse gases are key factors in global warming. The concentration of carbon dioxide emissions in the atmosphere has continued to increase in recent decades, mainly due to rapid population growth, biomass burning, land use change, deforestation, and environmental pollution [4]. Without restraint, by 2030 the global carbon emissions will be 30% higher than those in 2010 [5]. An increase in temperature will lead to a series of problems such as melting glaciers and rising sea levels. Then, the settlement of human beings will be affected. Greenhouse gas emission reductions—including CO_2_, SO_2_, etc.—are very important, which is not only an appeal of the current era, but also a strategic requirement for sustainable development.

Compared with industrialized countries, carbon dioxide emissions from energy consumption in newly industrialized countries have distinctly increased since the 1990s [6]. According to statistical data, since 2007 the carbon emissions in China have exceeded those in the USA, and China has become the world’s largest contributor of carbon emissions [7]. Under international pressure to reduce carbon emissions and domestic pressure to improve environmental quality, China is compelled to implement energy saving, emission reduction, and low carbon development. To shoulder its responsibility as one of the world’s great countries, China proposed ambitious environmental goals in 2014: carbon emissions will peak around 2030 [8,9,10], carbon intensity will decrease 60–65% compared with that in 2005 [11,12,13,14], and non-fossil energy will account for about 20% of primary energy consumption [15]. Moreover, during the 13th Five-year Plan (2016–2020), China promised to actively control carbon emissions and implement emission reduction, and quickened the pace of developing a low carbon economy by implementing far reaching policy measures [16]. China’s economy is entering the ‘new normal’, and the Chinese government proposed the establishment of ecological civilization. Against such backgrounds, the carbon emission reduction and control measures not only could hinder China’s economic development, but also provide challenges for China’s industrialization and urbanization in the development stage.

To realize the targets for energy conservation and emission reduction as soon as possible, China designated 36 cities (Beijing, Shanghai, Qingdao, etc.) as pilot areas for low carbon measures in 2010 and 2011 [11]. As a consequence, the relevant government departments and organizations were required to actively cooperate with each other to complete the program. One of the important missions in a province is to analyze the driving factors of carbon emissions and slow the growth rate of carbon emissions fundamentally, thereby constructing a low carbon economy. Shandong Province is China’s third-largest economic province behind Guangdong and Jiangsu, and accounts for 10% of China’s total energy consumption dominated by fossil energy [17,18]. Moreover, the carbon emissions in Shandong Province are the largest nationally [19]. Driven by the rapid development of urbanization, in 2015, Shandong Province’s population scale ranked the second nationally, of which the urban population comprised 57%. Based on these characteristics, Shandong Province is selected as the research object in this study for the exploration of the factors driving carbon emissions.

This study attempts to comprehensively explore the driving factors of carbon emissions and deeply investigate the impact of urbanization on carbon emissions in the case of Shandong Province in China. The results can help policy makers to formulate corresponding energy conservation and emission reduction policies more accurately.

The motivations of exploring carbon emission drivers from the perspective of carbon density are as follows. First, traditionally, the driving factors for decomposing carbon emissions are mostly similar, which results in that it is difficult to introduce other new drivers. In addition, the impact of urbanization on carbon emissions is gradually increasing. It is not enough to only consider the effect of population urbanization on carbon emissions. Therefore, the urbanization effect is more specifically characterized by population factor, traffic congestion, and land urbanization, which can be obtained by decomposing carbon density. Furthermore, carbon density is the ratio of carbon emissions to administrative area. Hence, the driving factors of carbon emissions can be well studied, because the administrative area is basically unchanged.

This study extends the previous literature in the following ways. (1) Instead of total carbon emissions, per capita carbon emissions, and carbon emission intensity, we explore the drivers of carbon emissions from the perspective of carbon density. (2) Based on structural change view, 15 influencing factors are evaluated; therefore, the driving factors of carbon emissions are described more comprehensively than previous studies. New driving factors including traffic congestion and land urbanization are introduced in terms of the extended Kaya identity, and specially the effect of the urbanization on the carbon emissions is investigated in detail.

**Figure 1:**
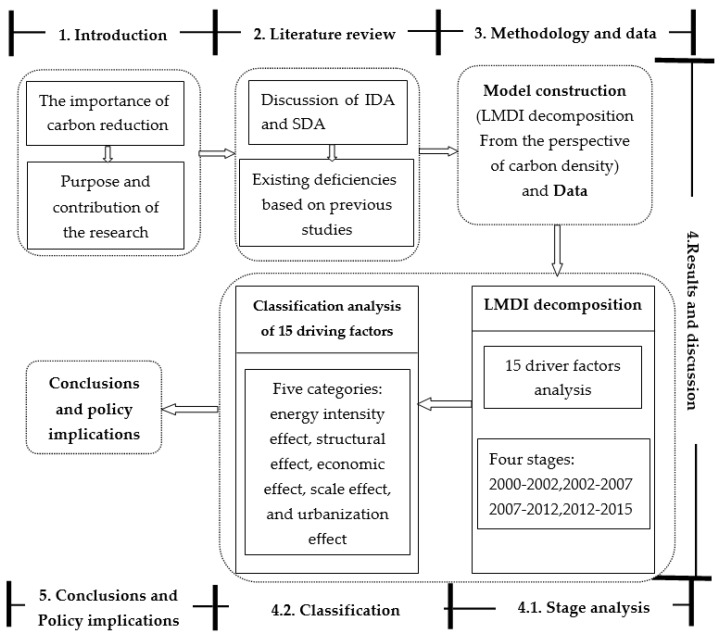
Framework of this research.

As shown in Figure 1, an integrated framework provides the clear research path of this study. Given that Shangdong Province’s goals with respect to energy conservation and emission reduction, this study aims to employ LMDI to investigate the driving factors from the perspective of carbon density. To explore the periodical changes of drivers, we divide the study interval into four stages based on the annual growth rate of carbon density. Then, in terms of the properties, 15 driving factors are classified as five categories. Accordingly, the contribution of each effect to carbon density is got. Finally, we present the conclusions and policy implications.

This study is organized as follows. Section 2 provides the related literature reviews. We describe methodology and related data in Section 3. Section 4 presents and discusses the decomposition results. Section 5 shows policy implications. Finally, we conclude this study.

## 2. Literature Review

The relationship between economic growth and energy use, as well as economic growth and environmental pollution, has been the topic of several research projects in the past few years. Magazzino employed a panel VAR technique to study the relationship among carbon emissions, energy consumption, and economic growth in 10 Middle East countries from 1971 to 2006 and found that the response of economic growth to CO_2_ emissions was negative in the estimated coefficients and impulse responses in six GCC countries [20]. Jalil and Mahmud employed an autoregressive distributed lag model (ARDL) approach to find that carbon emissions are mainly determined by income and energy consumption in the long run [21]. Alam et al. found that China supported the environmental Kuznets curve hypothesis only in the long run [22]. The objective of this paper is to achieve a win–win situation of reduction in carbon emissions and growth in economy. Consequently, Shandong Province must precisely recognize the factors driving carbon emissions, and adopt the appropriate policy to address each factor under the premise of stabilizing the economy.

Chinese and international research about the driving factors of carbon emissions can be divided into two types: structural decomposition analysis (SDA) modeling and index decomposition analysis (IDA) modeling. Mi et al. employed SDA based on a multiregion input–output model to investigate the determinants of changes in China’s export-embodied carbon emissions. It concluded that the changing structure of Chinese production was the primary cause of decline in Chinese export-embodied CO_2_ emissions after 2008 [23]. Su et al. studied the drivers of emission changes in Singapore using SDA, and found that the expansions of export-oriented industries and export volume caused emissions to increase, and fuel switching and energy efficiency helped reduce emissions growth [24]. Therefore, based on the SDA decomposition, the carbon emissions flow included in import and export can be explored [25,26,27,28]. Llop employed the demand-driven input–output model, as well as a simple method, to decompose the changes of energy gross output into different determinants. The results showed that the technology contributed positively to increasing energy output, while the contribution of final energy demand was negative [29]. Although SDA can be employed to study energy and carbon emissions related issues, there are still some problems. On the one hand, from an economic perspective, the factors influencing carbon emissions can be explored using SDA based on inputs and outputs [30]. Thus, the SDA approach is closely related with the economic system, and the resulting conclusions mainly apply to energy saving and emission reduction in terms of ‘supply–demand’ functions. On the other hand, because input–output data are updated rather infrequently, intensive and timely SDA studies are difficult.

In contrast to SDA, IDA has its own advantages. First, IDA originates from the energy analysis. From an energy perspective, IDA has a stronger connection with the energy system in the exploration of factors influencing carbon emissions based on comprehensive data [31]. Second, IDA can examine individual driving factors in more detail and in greater depth on a macro-scale. The IDA approach also can help the decision-makers identify the main driving factor on a macro-scale and formulate targeted policy. Third, IDA has only a modest data requirement; thus, differences in carbon emissions at different times and in different regions can be analyzed and compared easily [32]. Furthermore, compared with SDA, IDA is more flexible, simpler, and more accessible, and the forms of analysis can be varied. In this study, the factors driving the carbon emissions of final energy consumption in Shandong Province are explored by using IDA.

The logarithmic mean Divisia index (LMDI) method is widely utilized in IDA. Furthermore, compared with the other types of index decomposition analysis, LMDI can easily address the problems about the zero value in the decomposition [33] and of partial incomplete data. Therefore, the decomposition analysis using LMDI is a preferred method compared to others [34,35].

In terms of the Kaya identity [36,37], the LMDI decomposition was used to investigate the driving forces of GHG emissions in some previous literature [38,39,40,41]. Du and Lin employed LMDI to analyze the change of carbon emissions from 1991 to 2014 in China, and found that three main factors—labor productivity, energy intensity, and industry size—influenced the carbon dioxide emissions of China’s metallurgy industry [42]. Jiang et al. studied provincial-level carbon emission drivers and emission reduction strategies in China by using combined multi-layer LMDI decomposition with hierarchical clustering. The results showed that provincial economic expansion and energy intensity reduction have played a leading role in increasing and reducing carbon emissions, respectively [43]. Above all, the conclusions are different due to the differences in the subjects and the variability of the Kaya identity. However, most studies decomposed carbon emissions and found that energy intensity, economic output, and population scale were the major driving factors [44,45,46,47]. As China’s industrialization and urbanization advance, cities have become important areas of producing CO_2_ emissions. The research on the effect of urbanization on CO_2_ emissions is of importance for understanding the driving impacts of urbanization, evaluating the emission reduction task, and providing the scientific basis for low-carbon urbanization [48]. The driving factors often identified in previous studies have been not able to completely reflect the internal causes for the growth of carbon emissions. Thus, when studying the factors that drive carbon emissions, urbanization is gradually being taken into consideration by some scholars.

Ye et al. [49] divide nine departments into seven economic sectors and two “life sectors” through the Kaya identity, and then investigate the urbanization factor in China. However, this analysis is only based on the proportion of urban population in the total population. In most studies that examine the effect of urbanization on carbon emissions, only population urbanization has been used to represent urbanization [13,50,51,52], which is not all-inclusive. Urbanization is a process of coordinated expansion of population and development of land. Therefore, the results from investigations that focus only on population urbanization are far from optimal. Recently, a large number of scholars researching carbon emissions have begun also to indirectly consider land urbanization using other quantitative methods [53,54,55,56]. Nevertheless, there is no research that has considered land urbanization directly as a driving factor of carbon emissions based on factor decomposition. That is to say, no research has examined the direct causal relationship between land urbanization and carbon emissions. Therefore, in this study, from the perspective of carbon density (defined as the proportion of carbon emissions in an administrative area), land urbanization and traffic congestion are introduced to enrich the analysis of urbanization effect on carbon emissions, and thus investigate the urbanization effect more deeply.

Although the results from existing research play significant roles in formulating the carbon reduction policies in Shandong Province, there are still some deficiencies. First, as the economy increases and urbanization accelerates, the impact of urbanization on carbon emissions is becoming more serious. It is incomplete to only represent the effect of urbanization on the carbon emissions by population migration. Moreover, the current explanation of factors that affect carbon emissions is not adequate, and some hidden key factors are always neglected. Second, most of the literature for carbon emissions focuses on exploring the variations in each factor during the sample period. However, there is little research exploring the periodic change of factors. Finally, previous studies have only investigated a small number of driving factors; thus, some key influencing factors are obscured or omitted, which makes effective decision-making difficult.

## 3. Methodology and Data

### 3.1. Model Construction

Previous literature has mainly raised the discussion about the decomposition of carbon emissions, leading to similar results but with minor differences. Apart from these previous studies, in this study, from the view of carbon density based on the extended Kaya identity, the carbon emissions due to the final energy consumption are decomposed by using a “two-layer complete decomposition method” in this study. The model is represented by Equation (1).

(1) $$\begin{array}{c} CD=\frac{C}{S}=\frac{\sum_{ij}C^{ij}}{S}=\sum_{i=1}^{4}\sum_{j=1}^{3}\frac{C_{}^{ij}}{F_{}^{ij}}\times \frac{F_{}^{ij}}{F_{}^{i}}\times \frac{F_{}^{i}}{Y_{}^{i}}\times \frac{Y_{}^{i}}{Y}\times \frac{Y}{P}\times \frac{P}{S}+\\ \sum_{i=5}\sum_{j=1}^{3}\frac{C_{}^{ij}}{F_{}^{ij}}\times \frac{F_{}^{ij}}{F_{}^{i}}\times \frac{F_{}^{i}}{TD_{}}\times VTD\times {\left(\frac{S_{\mathrm{load}}}{VN}\right)}^{-1}\times \frac{S_{\mathrm{load}}}{S}+\\ \sum_{i=6}\sum_{j=1}^{3}\frac{C_{}^{ij}}{F_{}^{ij}}\times \frac{F_{}^{ij}}{F_{}^{i}}\times \frac{F_{}^{i}}{AUI}\times \frac{AUI}{UP}\times \frac{UP}{S}+\\ \sum_{i=7}\sum_{j=1}^{3}\frac{C_{}^{ij}}{F_{}^{ij}}\times \frac{F_{}^{ij}}{F_{}^{i}}\times \frac{F_{}^{i}}{TRI_{}}\times \frac{TRI_{}}{RP}\times \frac{RP}{S} \end{array}$$

Equation (1) can be further expressed as Equation (2).

(2) $$\begin{array}{c} \frac{C}{S}=\sum_{i=1}^{7}\sum_{j=1}^{3}\frac{C^{ij}}{S}=\sum_{i=1}^{4}\sum_{j=1}^{3}CEC^{ij}•EM^{ij}•EIP^{i}•ES^{i}•PCG•PD\\ +\sum_{i=5}^{}\sum_{j=1}^{3}CEC^{ij}•EM^{ij}•EIT•ATD•{\left(TC\right)}^{-1}•LU\\ +\sum_{i=6}^{}\sum_{j=1}^{3}CEC^{ij}•EM^{ij}•EIU•AUI•UPD+\sum_{i=7}^{}\sum_{j=1}^{3}CEC^{ij}•EM^{ij}•EIR•ARI•RPD \end{array}$$

In Equations (1) and (2), *i* represents each of the seven sectors, respectively: agriculture, forestry, animal husbandry, and fishery (abbreviated as ‘agriculture’); industry; construction; commerce; transport, storage, and post (abbreviated as ‘transport’); urban residential consumption; and rural residential consumption. Likewise, *j* is *j*-th energy consumption in each sector (total coal, total oil, and natural gas), respectively. The correlated variables are described in Table 1.

Energy consumption of the three types of energy is divided according to “China Energy Balance Sheet” [57]. Furthermore, the final energy consumption sectors are also apportioned among agriculture, industry, construction, commerce, transport, urban residential consumption, and rural residential consumption. Commerce includes wholesale, retail trade, hotels, restaurants, and others.

Using the LMDI method, carbon density is decomposed into 16 driving factors: carbon emission coefficient (CEC), energy mix (EM), energy intensity of production (EIP), economic structure (ES), per capita gross domestic production (PCG), population density (PD), energy intensity of transportation (EIT), average transport distance (ATD), traffic congestion (TC), land urbanization (LU), energy intensity of urban residents (EIU), average urban income (AUI), urban population density (UPD), energy intensity of rural residents (EIR), average rural income (ARI), and rural population density (RPD).

Equation (2) can be also expressed as Equation (3).

(3) $$\begin{array}{c} \Delta CD=CD^{T}-CD^{0}=\Delta CD(CEC)+\Delta CD(EM)+\Delta CD(EIP)+\Delta CD(ES)+\Delta CD(PCG)\\ +\Delta CD(PD)+\Delta CD(EIT)+\Delta CD(ATD)+\Delta CD(TC)+\Delta CD(LU)+\Delta CD(EIU)\\ +\Delta CD(AUI)+\Delta CD(UPD)+\Delta CD(EIR)+\Delta CD(ARI)+\Delta CD(RPD) \end{array}$$

In Equation (3), $\Delta CD(X_{k})$ represents the contribution of the *k*-th driving factor of carbon density in the range of [0, T]. According to the derivation of Appendix A, the contribution of each driving factor can be obtained. Among them, the combined contribution of 15 driving factors can be calculated through Equation (4).

(4) $$\Delta CD(X_{k})=\sum_{i}\sum_{j}\omega_{ij}(t^{*})\ln\frac{X_{k}^{ijT}}{X_{k}^{ij0}}\;$$

The calculation for the contribution of traffic congestion is described by Equation (5).

(5) $$\Delta CD(X_{k})=\sum_{i}\sum_{j}\omega_{ij}(t^{*})\ln\frac{X_{k}^{ij0}}{X_{k}^{ijT}}\;$$

In Equations (4) and (5),

(6) $$\omega_{ij}(t^{*})=L(CD^{ijT},CD^{ij0})=\{\begin{array}{c} \left(CD^{ijT}-CD^{ij0}\right)/(\ln CD^{ijT}-\ln CD^{ij0}),CD^{ij0}\neq CD^{ijT}\\ CD^{ij0},CD^{ij0}=CD^{ijT} \end{array}$$

### 3.2. Data

Except carbon emission data, the data are all derived from China Statistical Yearbook [58], China Energy Statistical Yearbook [57], and Shandong Statistical Yearbook [59]. The gross domestic product (GDP), outputs of each department and resident’s income are converted to constant prices in 2000. The data for total coal and total oil and natural gas published in Chinese Energy Statistical Yearbook are selected to measure carbon emissions of final energy consumption in Shandong Province by using Equation (7).

(7) $$C=\sum_{i}\alpha_{i}F_{i}\;$$

In Equation (7), $\alpha_{i}$ represents the carbon emission coefficient of the *i*-th energy consumption, and $F_{i}$ is the *i*-th energy consumption (standard coal). The carbon emissions coefficients of all consumption are taken from Hu and Huang [60] and IPCC [61].

## 4. Results and Discussion

The carbon emission coefficients are assumed to be constant during the research period, and the contribution rate is zero. Thus, the effect of carbon emission coefficients could be neglected when analyzing the driving factors.

### 4.1. Stage Analysis

**Figure 2:**
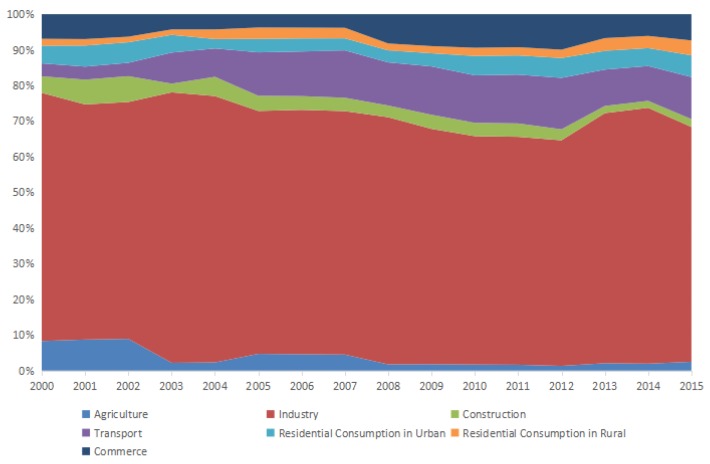
Carbon density of each sector.

During 2000–2015, the carbon density increased from 693.13 tons/km^2^ (t/km^2^) to 1968.88 t/km^2^ in Shandong Province, following an annual growth rate of 7.21%. As shown in Figure 2, from 2000 to 2015, the proportion of carbon density in Industry fluctuated, and decreased by 3.76%. Though the proportion of carbon density of agriculture changed slightly, as a whole, it showed a decreasing trend (from 8.30% to 2.47%). For construction, the proportion of carbon density also changed relatively little, and only decreased by 2.44%. Transport exhibited the greatest variation in the proportion of carbon density it contributed, which increased by 8.18% overall. For urban residential consumption and rural residential consumption, the proportions of carbon density both fluctuated and followed an increasing trend, but the change rate of rural residential consumption was higher than urban residential consumption. For commerce, carbon density increased overall.

In brief, although the contribution of Industry decreased to some extent, it still accounted for 65.89% and was the main contributing sector to carbon density. Transport gradually increased its proportion in the overall carbon density, and was the second largest contributing sector. The proportion of carbon density contributed by agriculture gradually declined, and agriculture and construction made the smallest contributions to carbon density. The contribution to carbon density by urban residential consumption was 1.90% higher than rural residential consumption, but the variation was smaller.

**Figure 3:**
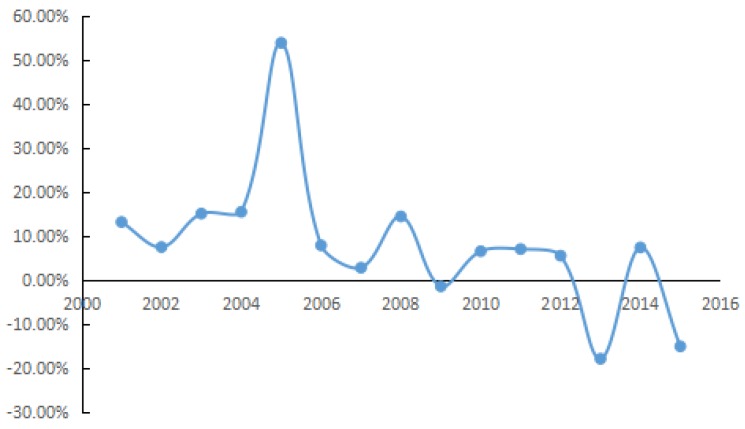
Growth rate of carbon density from 2000 to 2015 in Shandong Province.

The annual growth rate of carbon density in Shandong Province during 2000–2015 is presented in Figure 3. During 2000–2002, there was a slight decreasing trend, with an annual decline rate of 10.38%. From 2002 to 2007, the growth rate of carbon density fluctuated more widely than in later years, following an annual growth rate of 17.88%. During 2007–2012, carbon density increased with an average annual rate of 6.39%. From 2012 to 2015, carbon density fluctuated around a decreasing trend, and the average annual growth was −9.09%. In short, the growth of carbon density fluctuated over time and exhibited a short-term wave effect with periodicity in Shandong Province. Most previous literature selects fixed interval for study, in this way, fluctuations in carbon density are obscured. In addition, previous research neglects some important details, including the variation in the key factors causing the turning points. Therefore, to deeply explore the variation trend of the factors driving the carbon density, the study period (2000–2015) in Shandong Province is divided objectively according to the annual average growth rates just presented. This division results in four stages: 2000–2002, 2002–2007, 2007–2012, and 2012–2015. Moreover, the main driving factors and the turning points in the different stages also are examined.

**Figure 4:**
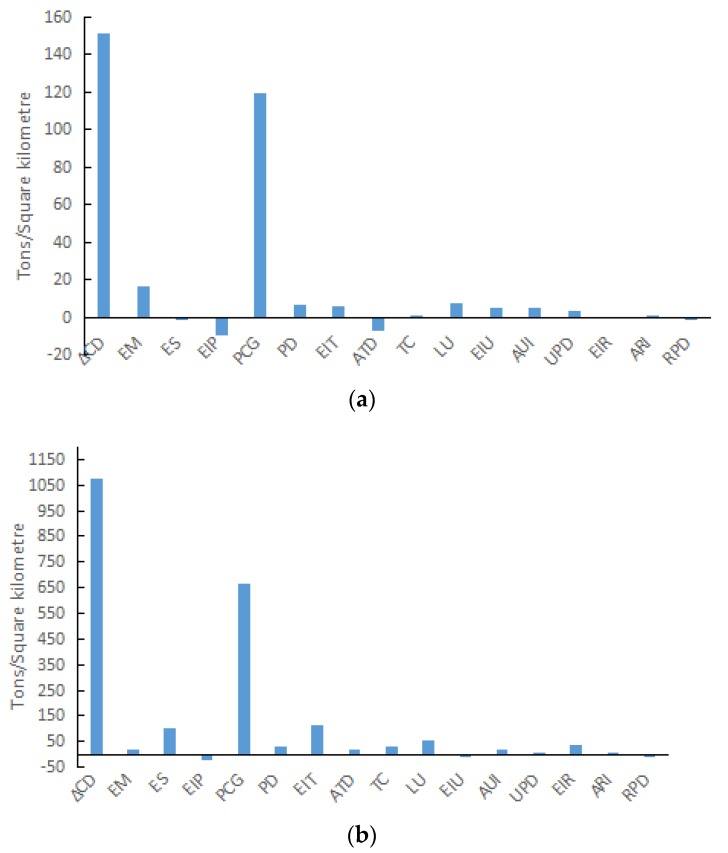

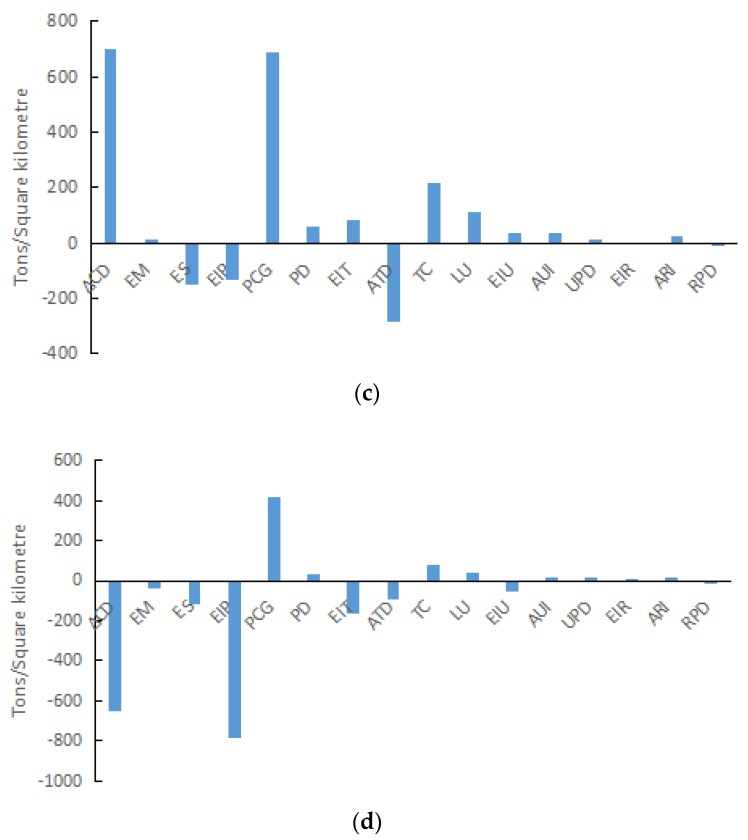
Contribution of each factor during different periods. (**a**) Stage one (during 2000–2002); (**b**) Stage two (during 2002–2007); (**c**) Stage three (during 2007–2012); (**d**) Stage four (during 2012–2015).

In Shandong Province, carbon density increased in each of the first three stages, but declined in the fourth stage. The decomposition results are shown in Figure 4. In the first stage (2000–2002), the positive driving factors of carbon density included energy mix, per capita GDP, population density, energy intensity of transportation, traffic congestion, land urbanization, energy intensity of urban residents, average urban income, urban population density, and average rural income. The negative driving factors were economic structure, energy intensity of production, average transport distance, energy intensity of rural residents, and rural population density. Per capita GDP and energy mix were the first and second most positive driving factors, respectively, and energy intensity of production and average transport distance were the first and second most negative driving factors, respectively.

In the second stage (2002–2007), carbon density increased substantially from 844.54 t/km^2^ to 1922.19 t/km^2^. Per capita GDP remained the main contributing factor, following an annual growth rate of 13.37%. The rapid growth of Chinese economy in this period resulted in a huge amount of energy consumption, and stimulated carbon density to reach 666.33 t/km^2^. The contribution of energy intensity of transportation increased from 2.70% in the previous stage to 15.87%. This increase in the energy consumption by the transportation sector led to energy intensity of transportation as the second largest positive driving factor (replacing energy mix). Furthermore, economic structure changed from a negative driving factor in the first stage into a positive driving factor in the second stage. The main reason for this change is that the proportion of Industry in Shandong’s total GDP increased from 43.98% to 51.53%, and Industry became the main commercial consumer of energy. The increased proportion of Industry resulted in increased energy consumption, and thus increased carbon density by 101.23 t/km^2^. Among the negative driving factors, energy intensity of production only contributed −1.85%, which did not restrain the increase in carbon density induced by economic growth. Compared to the previous stage, energy intensity of urban residents became a negative driving factor, and energy intensity of rural residents and average transport distance became positive driving factors.

In the third stage (2007–2012), the growth rate of carbon density slowed and the total carbon density increased by 697.99 t/km^2^. Overall, the negative driving factors contributed minus 75.62% to carbon density. The contribution of average transport distance accounted for −49.02%, which was the leading negative driving factor. The economic structure of production sector driving carbon density changed from a positive factor in the previous stage to a negative factor. This change was mainly because the proportion of industry in Shandong’s GDP reduced to 45.58%, which decreased the contribution of industry to carbon density from 751.75 t/km^2^ in the previous stage to 345.49 t/km^2^ in the third stage. The inhibiting effect of energy intensity of production on carbon density increased. Besides that, in commerce, the energy intensity in the other three sectors all declined. As a result, the contribution of energy intensity of production was −18.76%, and it was the third largest negative driving factor. The growth rate of per capita GDP also slowed to some extent (8.20%), but it was still the largest positive contributing factor to carbon density. The promoting effect of traffic congestion and land urbanization also increased, making them become the second and third most important contributing factors, respectively. The main reason for these changes was the growth in the living standard; consequently, the amount of civil car ownership increased with an annual growth rate of 23.36%. In 2012, the proportion of civil car ownership in Shandong Province accounted for 9.39% of the total amount nationwide, which ranked second and only followed Guangdong Province. In addition, the average annual growth rate of road area was 7.52%.

In the fourth stage (2012–2015), carbon density presented a step function decline. Compared with that in the first three stages, the inhibiting effect of energy intensity of increased rapidly and became the most important negative driving factor. The inhibiting effect was much greater than that of the other negative driving factors. The reason was that energy intensity of industry, construction, and commerce declined significantly. Only carbon density of Industry decreased by 361.37 t/km^2^. Energy intensity of transportation changed from being a positive driving factor into the second largest negative driving factor. Energy consumption in transport decreased from 29.1651 million tonnes in 2012 to 17.9833 million tonnes in 2015. Compared with that in the previous stage, energy intensity of urban residents changed from a positive driving factor to a negative driving factor. The growth rate of per capita GDP tended to be stable, and the promoting effect of this factor on carbon density slowed. The positive driving effect of traffic congestion and land urbanization slowed to some extent, nevertheless, these remained the second and third most important contributing factors, respectively.

### 4.2. Classification Analysis of Driving Factors

**Figure 5:**
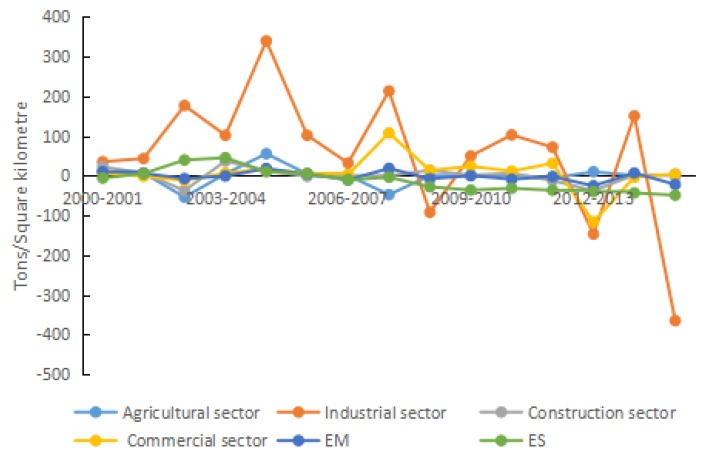
Contribution of each factor in structural effect.

**Figure 6:**
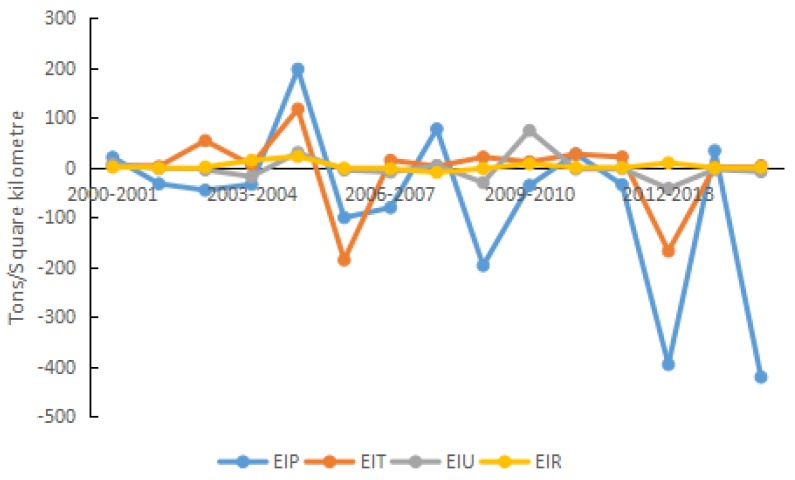
Contribution of each factor in energy intensity effect.

**Figure 7:**
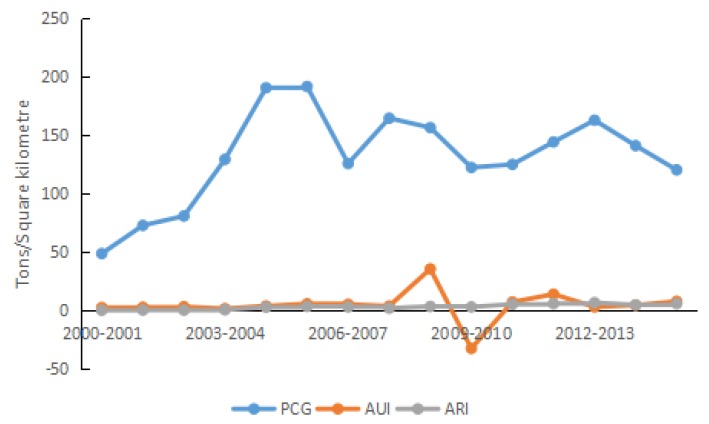
Contribution of each factor in economic effect.

**Figure 8:**
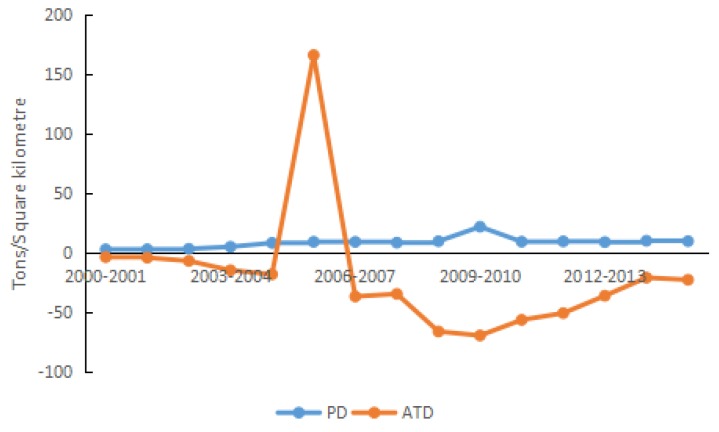
Contribution of each factor in scale effect.

**Figure 9:**
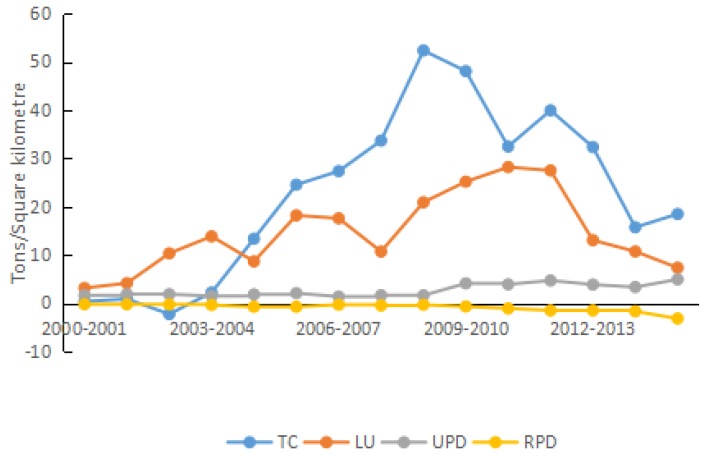
Contribution of each factor in urbanization effect.

In terms of the properties of factors, the 15 driving factors are divided into five categories, i.e., energy intensity effect, structural effect, economic effect, scale effect, and urbanization effect. The classifications are presented in Table 2, and the effect analysis is plotted in Figure 4, Figure 5, Figure 6, Figure 7, Figure 8 and Figure 9.

#### 4.2.1. Structural Effect

The energy consumption of seven sectors in Shandong Province is divided into total coal, total oil, and natural gas. The emission factor for these three energy sources is: total coal > total oil > natural gas. Therefore, an increase in the proportion of energy consumption with high carbon emission factor will drive carbon density. Thus, the contribution of energy mix to carbon density is manifested in the consumption of various energy sources. That is, for any given level of total energy consumption, the larger is the proportion of coal in total energy consumption, the greater is the amount of CO_2_ generated.

During 2000–2015, the proportion of total coal fluctuated downward. However, coal consumption is still the biggest contributor to the increased carbon emissions in Shandong, which is basically the same to the result obtained by Wang et al. [62]. In other words, the coal-based pattern of energy consumption in Shandong Province is difficult to change in the short term. As a result, the contribution of energy mix to carbon density also fluctuated with both positive and negative variations, but overall the variation was negative (see Figure 5). The accumulative contribution to carbon density was −21.27 t/km^2^, and the contribution rate was −1.67%. These results indicate that, in recent years, China has achieved a certain reduction in the proportion of coal in its total energy consumption. However, the development pattern in Shandong Province is still extensive. It is difficult to change the production mode of energy dominated by coal in a short term. Therefore, the impact of energy mix effect on the reduction of carbon density was not apparent during the study period in Shandong Province. This finding is consistent with [63], though the research provinces and years are different. Energy mix has not played an important role in the reduction of carbon emissions in Shandong Province. This analysis suggests that the reduction in the consumption of fossil energy (especially coal), the positive development of new energy and the vigorous promotion of clean energy (such as wind energy, hydro-energy, and solar energy) can have a certain inhibiting effect on the increase of carbon intensity in Shandong Province.

During 2000–2015, energy consumption in agriculture, industry, construction, and commerce accounted for 3.11%, 64.68%, 3.94%, and 6.93% of total energy consumption, respectively. Thus, industry was by far the sector with the maximum energy consumption. Any increase in the proportion of industrial output would be accompanied by the consumption of huge amounts of fossil energy, resulting in the increase of CO_2_ emissions. During 2000–2015, the proportion of agriculture gradually decreased from 15.22% to 8.23% in Shandong Province. The share of Industry followed an “inverted U-shaped” downward trend, and decreased from 43.97% to 41.13%. The proportion of construction fluctuated, and decreased from 5.98% to 5.80%. The ratio of commerce increased from 28.30% to 41.32%.

From 2000 to 2015, economic structure reduced carbon density by 179.65 t/km^2^, and the contribution rate was −14.09%. In the early years of 2000–2002 in Shandong Province, the accumulative contribution of economic structure to carbon density was small (only −1.60%). This limited influence was mainly because there was no change in the proportion of Industry, and the proportions of both construction and commerce increased. During 2002–2005, the contribution of economic structure to carbon density increased continuously, and peaked in 2005. This was because the proportion of industrial output increased significantly during this period, and tended to be stable during 2005–2008. After 2005, the negative driving effect of economic structure on carbon density was enhanced remarkably, due to not only the reduction in the proportion of industry, but also the increases in the proportion of commerce and the technological progresses of each sector. Therefore, in comparison to energy mix, economic structure has greater potential for reducing carbon density. Reducing dependence on industry and enhancing development of the tertiary industry can effectively slow the increase of carbon density in Shandong Province.

#### 4.2.2. Energy Intensity Effect

Although the energy intensity of production in Shandong Province fluctuated, this factor was the primary inhibiting factor on carbon density, and could effectively slow the increase of carbon density. Wang et al. decomposed the influencing factors of energy-related carbon emissions over 1995–2011 in Shandong Province using the LMDI method [64]. The decomposition result of the energy intensity is consistent with our result, although the research period are different. During 2000–2015, the energy intensity of agriculture, industry, construction, and commerce followed a downward trend on the whole, and declined by 61.40%, 38.72%, 62.83%, and 58.11%, respectively. The accumulative contribution rate of energy intensity of the production sector to carbon density was −80.13%.

Although the energy intensity of production in Shandong Province fluctuated (see Figure 6), this factor was the primary inhibiting factor on carbon density, and could effectively slow the increase of carbon density. During 2000–2015, the energy intensity of agriculture, industry, construction, and commerce followed a downward trend on the whole, and declined by 61.40%, 38.72%, 62.83%, and 58.11%, respectively. The accumulative contribution rate of energy intensity of the production sector to carbon density was −80.13%.

However, in the early period of 2000–2002, the energy intensity of production in Shandong Province had less of an inhibiting effect on carbon density than in the subsequent years. The effect was limited because although the energy intensity of agriculture, construction, and commerce increased. The maximum energy consumption occurred in industry, whose energy intensity accounted for a larger proportion of the contribution to carbon density in the total production sector. During this period, the energy intensity of industry decreased only slightly. Thus, the negative driving contribution rate (i.e., inhibitory effect) of energy intensity from the production sector to carbon density was less than 10%. After 2005, successive five-year plans guided reductions in energy intensity. Energy intensity decreased by 20% and 16% as required by the “11th Five-Year Plan” and the “12th Five-Year Plan”, respectively. In addition, the carbon emission intensity decreased by 17% in the “12th Five-Year Plan”. Except for a tiny fluctuation in the energy intensity of commerce in Shandong Province, the energy intensity of the other three sectors all decreased.

Therefore, during 2012–2015, the accumulative contribution of energy intensity from the production sector to carbon density reached minus 119.96%, and the positive (i.e., stimulatory) effect of per capita GDP on carbon density was eliminated effectively, directly leading to the downward trend of carbon density. During 2000–2015, in Shandong Province, the contribution of energy intensity from the production sector to carbon density was negative (i.e., inhibitory) in most years. This impact was caused by the increase of energy intensity in industry, which suggested that the energy intensity of industry was the most influential factor affecting changes in carbon density.

In Shandong Province, the growth rate of the transit line length was smaller than growth rate of energy consumption in transport; thus, the energy intensity of transportation had a positive driving effect on carbon density, otherwise, it had a negative driving effect. During 2000–2015, the energy intensity of transportation increased from 24.10 to 68.26 tons of standard coal per kilometer. Overall, energy consumption exhibited an increasing trend, although there appeared a temporary downward trend in 2006 and 2013; as a result, the contribution to carbon density changed from positive to negative. There are two major causes. The first is that the energy consumption of transport increased by only 10.99%, while the transit line length increased by 155.72%, which was much larger than the growth rate of energy consumption in 2006 in Transport. The other is that the energy consumption decreased to a lower level temporarily in 2013, which is an outcome of signing of the “Memo for Jointly Advancing Construction of a Green Transport System in Shandong” between Ministry of Transport and Shandong Province, and the beginning of creating a green transport province. During 2000–2015, the accumulative negative (i.e., inhibitory) effect on carbon density reached minus 72.54 t/km^2^ on the whole, and the average contribution rate was −5.69%.

#### 4.2.3. Energy Intensity of Urban and Rural Residential Consumption

During 2000–2015, the energy intensity of urban residential consumption in Shandong Province fluctuated, but the variation was smaller than that of energy intensity of rural residential consumption. The energy intensity of urban residential consumption declined from 0.102 to 0.091 tons of standard coal per 10,000 yuan. The accumulative contribution rate to carbon density was negative (−1.25%) and smaller than that of rural residential consumption. In 2005 and 2010, a rising trend appeared in the energy intensity of Urban residential consumption; thus, the contribution of this factor to carbon density had two peaks (30.01 t/km^2^ and 74.16 t/km^2^). Overall, the energy intensity of rural residential consumption exhibited an increasing trend, and increased from 0.060 to 0.180 tons of standard coal per 10,000 yuan, following an annual growth rate of 7.61%. The accumulative carbon density driven by the energy intensity of rural residential consumption arrived at 34.42 t/km^2^, with an accumulative contribution rate of 2.70%.

After 2005, the energy intensity of rural residential consumption was always higher than that of Urban residential consumption. As the energy demand of the resident life sector increased, the utilization efficiency of energy by urban residents improved, and always exceeded that in rural areas. Moreover, there was no rising trend. Therefore, there was great potential for the reduction of carbon density by decreasing the energy intensity of resident life in Shandong Province. The decrease in the energy intensity of urban residential consumption had a great inhibiting effect on carbon density. However, after 2013, this effect was always in a slow decline. Thus, the decrease of energy intensity of Rural residential consumption is the key to restraining the increase of carbon density. However, it is difficult to change the living habits and the rigid demand of residents for energy in a short time. Therefore, to unleash the potential of the inhibiting effect of energy intensity in resident life on carbon density, it is necessary to publicize low carbon lifestyles, and encourage the use of clean energy (such as solar energy and wind energy) in the long term.

#### 4.2.4. Economic Effect

Per capita GDP reflects not only the economic development, but also the average consumption capability and affluence. During 2000–2015, per capita GDP increased steadily at an average annual rate of 10.02%. This specified that people’s living standard improved continuously. The increase in purchasing capability resulted in increased demand for durable products such as automobiles and household appliances. This demand increased energy consumption in resident life and in production, and ultimately increased carbon density. During 2000–2006, Shandong Province was in the critical stage of the industrialization and urbanization development. In this stage, the province was still mainly experiencing high-energy consumption, high emissions, and extensive economic development. Therefore, carbon density increased in concert with increased economic development. In 2015, Shandong was in the latter half of its late period of industrialization. As a result, the rigid demand for the energy and raw material slowed.

During 2000–2015, per capita GDP was responsible for increasing carbon density by a total of 1977.63 t/km^2^, and the contribution rate was 155.16%. Thus, per capital GDP was by far the primary positive driving factor of carbon density (see Figure 7). Our results are consistent with Zhang et al.’s finding [65], therefore, the green economy is the key point of carbon emission reduction in Shandong Province. Carbon density is generated together with economic growth, so efforts to control carbon emissions must consider emission reduction and economic development simultaneously. Therefore, emission reduction is to be realized in the province without negatively affecting economic development, achieving the win–win development goal.

During 2000–2015, the average urban income in Shandong Province increased from 6489.97 yuan to 17,976.35 yuan, following an annual growth rate of 7.03%. The increase in urban income was responsible for increasing carbon density by 72.56 t/km^2^ in this period, and the contribution rate was 5.69%. The average rural income increased from 2659.20 yuan to 7368.50 yuan at the same rate as average urban income (7.03%). It suggested that the increase of per capita income in Shandong Province was relatively balanced. Carbon density increased by 52.02 t/km^2^ due to average rural income, whose contribution rate was 4.08%. The improvement of per capita income increased the demand for durable products, promoted the rise of energy consumption in the production sector, and ultimately increased carbon density. Although from the perspective of consumer, both driving factors had contributing effects on carbon density, the effects were different. Due to the availability of commodities, consumer income, and consumption habits, the urban residents in Shandong Province were responsible for most of the consumption. The rural residents comprised the ‘potential consumer’ group. Thus, the contribution rate of average urban income to carbon density was higher than that of rural resident incomes.

#### 4.2.5. Scale Effect

During 2000–2015, the population density increased steadily from 573 to 627 people/km^2^, following an annual growth rate of 0.604%. In 2015, the population density in Shandong Province was the fifth highest in China, resulting in great demand for energy, and thereby increasing carbon density. Population density had a positive (i.e., stimulatory) effect on carbon density throughout the period, increasing it by 133.42 t/km^2^ with the contribution rate of 10.47%. Shen et al. explored drivers of carbon emission in Beijing and also found population scale was always a driving factor for the increase of carbon emissions [66], which was in accordance with the above conclusion. The increase of population leads to an increase in energy demand, which in turn affects carbon emissions. In 2010, there appeared a temporary increase of the population density in Shandong Province. As a result, the contribution of population density to the carbon density appeared to peak (see Figure 8). Since then, as the Shandong Government implemented the “two-child” policy, the control of population growth has been restricted and its effect on carbon density has been limited. Through education and publicity, the population’s understanding of environmental issues can be improved. Accordingly, the low carbon consciousness is to be cultivated and the habit of low carbon lifestyle is to be formed, thereby reducing the effect of population increase on carbon density.

Whenever the growth rate of the number of vehicles was lower than the growth rate of transit lines in Shandong Province, average transport distance had a positive driving effect on carbon density; otherwise, average transport distance had a negative driving effect. The increased ownership of private automobiles was the main cause for the substantial increase in the number of vehicles. After 2013, the ownership of private automobiles in Shandong Province was always the highest in China, resulting in high demand for huge amounts of energy, especially oil-derived fuels.

During 2000–2015, the transit line length increased by 154.96%, following an annual growth rate of 6.44%. The number of vehicles increased by 1245.33%, with an annual growth rate of 18.92%. The average transport distance decreased by 72.30%, and carbon density decreased by 269.55 t/km^2^. The accumulative contribution of average transport distance to carbon density was −21.15%. In 2006, there was an abrupt (but temporary) increase (155.72%) in the transit line length. However, the vehicle ownership increased by only 10.99%; as a result, the contribution of average transport distance to carbon density turned from negative to positive.

#### 4.2.6. Urbanization Effect

The traffic congestion synthesizes two factors (urban road area and number of vehicles) that are attributed to the urbanization effect. When the growth rate of road area is larger than that of the number of vehicles, the contribution of traffic congestion to carbon density is negative; otherwise, the contribution is positive. During 2000–2015, the road area in Shandong Province increased by 271.62%. The traffic congestion reduced by 72.38%, with an annual growth rate of −8.22%. Before 2006, the contribution rate of traffic congestion to carbon density was smaller than in later years. After 2006, the traffic congestion was the second largest positive driving factor of carbon density (see Figure 9). On the one hand, as people’s income increased and the consumption concept changed, these resulted in a rapid increase in demand for automobiles, which then led to a huge amount of energy consumption, followed by the increase in carbon density. On the other hand, the increased traffic congestion reduced the average travel speed of most vehicles, not only leading to time waste, but also resulting in excessive fuel consumption and environmental pollution. Thus, the utilization efficiency of transport energy decreased, yielding unnecessary waste. In 2009, the contribution of traffic congestion to carbon density peaked, and later presented a stepped decrease.

During 2000–2015, the traffic congestion pushed carbon density by 339.24 t/km^2^, and the accumulative contribution rate was 26.62%. Therefore, in Shandong Province, the proportion of “new energy” automobiles that utilize new (i.e., clean) forms of energy should be enlarged, and the government can strive to decrease the growth rate of fossil-fueled automobiles. Furthermore, Shandong Province is also to accelerate appropriate urban road construction, widen the road area, optimize the signaling at intersections, and be more aggressive in adopting the “three-dimensional” transportation strategy to relieve the gradual increase of traffic loading per unit area.

Before 2012, the contributing effect of land urbanization on carbon density in Shandong Province presented a fluctuating increase; after 2012, the effect gradually decreased. During 2000–2015, the overall accumulative carbon density was increased by 219.18 t/km^2^ due to land urbanization, and the contribution rate was 17.20%. After 2010, land urbanization became the third largest positive driving factor of carbon density. Substantially, urbanization is a process of synchronous urbanization of both the population and land. Meanwhile, land urbanization is an important way to promote the urbanization development. As urbanization progresses, on the one hand, the increase in road area prompts the conversion of agricultural land to urban land at a faster rate, and the high-speed expansion results in inefficient land utilization. On the other hand, the expansion of urban road area facilitates free movement, and helps people visit each other more frequently. Thus, land urbanization increases energy consumption continuously, and leads to increased carbon density. Therefore, while developing economy, improving urban and rural incomes, and accelerating urbanization process, it is of great importance for the government to control increases in the associated carbon emissions, improve the land utilization efficiency, and ensure the quality of urbanization development.

The population urbanization is the ultimate goal of urbanization development. With the economic development in Shandong Province, rural population transferred into urban areas, resulting in a continuous increase of the urban population. The pressure on environmental quality continued to increase in the city as the urbanization level improved continuously. During 2000–2015, the urbanization rate increased from 37.94% to 57.01%. Cities are the primary sites for economic activity, and the increasing urban population cause energy consumption to increase. Thus, the migration of the rural population into urban areas increases carbon density. During 2000–2015, the contribution of urban population density to carbon density was continuously positive, although the growth range became smaller over time. Carbon density increased by 39.91 t/km^2^ due to urban population density, and the contribution rate was 3.13%. Nevertheless, the contribution of rural population density to carbon density was continuously negative. Carbon density decreased by 13.62 t/km^2^ due to rural population density, whose contribution rate was −1.07%.

From the perspectives of population, land urbanization, and traffic congestion, the urbanization effect increased carbon density continuously during 2000–2015 in Shandong Province. Ren et al. also found that the urbanization has distinct positive effects on energy consumption and carbon emissions during the process on urbanization in Shandong Province [18], but we have different definitions of urbanization. Ren et al. only considered population urbanization, however, this study completely considered the urbanization from three dimensions. From 2000 to 2010, the annual increase in carbon density due to the urbanization effect showed a stepped rising trend, and peaked in 2010. During 2010–2015, the effect declined year by year, although a temporary rise occurred. Overall, due to the urbanization effect, the accumulative carbon density increased by 584.70 t/km^2^ during 2000–2015, and the contribution rate was 45.87%.

**Figure 10:**
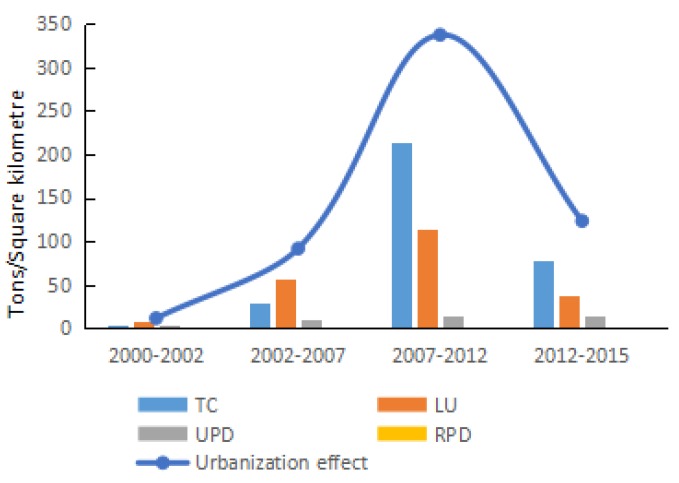
Contribution of each factor in urbanization effect during different periods.

Based on the stage analysis of carbon density (see Figure 10), the urbanization effect in all four stages promoted the increase of carbon density, but the magnitude of the effect was different among stages. In the third stage, the urbanization effect contributed the most to carbon density. In the first stage (2000–2002), the rural population density was higher than the urban population density. Because residents were the consumer side of energy consumption, the primary energy consumption took place in cities, which resulted in a lower contribution of the demographic factor in the overall urbanization effect to carbon density. In this stage, rural population slowly transferred to urban areas, and the contribution of rural population density to carbon density was negative, while the contribution of urban population density was positive. At the beginning of the study period, because of the modest living standard, limited availability of commodities and technical restrictions in Shandong Province, the desire to purchase durable goods such as private vehicles was weak, and therefore the positive effect of traffic congestion on carbon density was smaller than in later years. Due to extension of road area, land urbanization was the primary driving factor in the urbanization effect, but the contribution was also smaller than in later years, and the urbanization effect on carbon density was not obvious. The accumulative carbon density due to urbanization increased by 11.08 t/km^2^, and the contribution rate was 7.32%.

In the second stage (2002–2007), as the rural population in Shandong Province gradually transferred to urban areas, the contributions of urban and rural population density to carbon density were both greater than before 2002. Due to the continuous improvement of living standards and technological development, the demand for travel gradually increased. Thus, compared to the previous stage, the positive driving effect of traffic congestion on carbon density was greater and it became the second largest positive driving factor in the urbanization effect.

In the third stage (2007–2012), as industrialization and urbanization advanced, due to social development, the association among people was more frequent, and the number of private automobiles increased rapidly (185.65%). Meanwhile, Shandong Province intensified the construction of transportation infrastructure. As a result, traffic congestion became the largest positive driving factor in the urbanization effect, and the contribution almost doubled the land urbanization effect. In this stage, the contribution of the urbanization effect to carbon density peaked (48.33%).

In the fourth stage (2012–2015), Shandong Province progressed to the latter period of industrialization. Although the number of private automobiles and road area continuously increased, the overall energy consumption in the transportation sector decreased. These changes occurred due to the following reasons. On the one hand, as the environmental awareness of the public increased, and the government implemented a subsidy for automobiles powered by ‘new’ energy, the new energy automobiles became more commonplace. On the other hand, the relevant policies to perfect urban traffic infrastructure construction and reduce road congestion were introduced. In this stage, the contribution rate of urbanization effect on carbon density declined to 18.86%, while the traffic congestion was still the main contributing factor.

## 5. Policy Implications

Based on the above results, to enable Shandong Province to realize the goal of carbon emission reduction and a low-carbon economy construct, the following policy suggestions are provided for reference.

(1) It is of importance for Shangdong Province to optimize industrial structure. The high-carbon industry is mainly concentrated in the secondary industry, and the economic structure dominated by the secondary industry in Shandong Province is largely the main reason for the increase in carbon emissions. The government should optimize industrial structure continuously, promote the coordinated development of industry, and implement policy support and guidance. In other words, Shangdong Province should utilize its own industrial advantages, focus on developing the tertiary industry, reduce the proportion of the secondary industry in the economy, and give full play to the potential and advantages of the tertiary industry in low carbon development.

(2) There is a need to improve the quality of urbanization in Shandong Province. The government should control the growth in the number of automobiles, and encourage the development of ‘new energy’ automobiles, and continuously implement subsidies as a ‘never fall back mechanism’ for new energy automobiles. Meanwhile, it is vital to perfect the construction of transportation routes, reduce the duration of traffic congestion, and complete the rational planning and management of urban layouts. Furthermore, the government should adjust measures to local conditions and consider the coordination issues on the resources and environment in the layout of urban agglomerations. In order to slow down traffic congestion, the government could adopt the ‘three-dimensional’ transportation strategy to relieve the gradual increase of traffic loading.

(3) Shandong Province is to improve energy mix, and increase energy efficiency. Toward the end of the study period, Shandong Province was in the process of post-industrialization. Although the demand for fossil fuel in Shandong Province slowed over time, the total energy consumption remained high. Judging from energy mix analyzed in the previous section, Shandong’s energy is mainly based on coal, and the proportion of other fossil fuel is small. Therefore, the government can control the total coal consumption, vigorously develop coal purification and washing technology, and accelerate the transformation of energy mix from coal-based framework to clean energy and renewable energy. Meanwhile, there is a need to strengthen international cooperation, develop energy saving and emission reduction technology, and increase the efficiency of energy utilization, especially for industry and transport.

(4) It is crucial for Shandong Province to cultivate the consciousness of emission reduction and guide low carbon lifestyles. As the economy grows, the energy consumption by citizens gradually increases. Because improvements in the energy utilization efficiency of urban residents cannot effectively offset the increase in the carbon emissions due to population migration in Shandong Province, the focal point of carbon emission reduction associated with consumption has been the carbon capacity of residents. The government can guide the consumption patterns and lifestyles of residents, strengthen publicity about low carbon lifestyles, cultivate the consciousness of emission reduction, and promote habits for lifestyles in a low carbon economy. Furthermore, through publicity, residents’ attention to the environment can be increased, and then they can increase their willingness to accept new energy vehicles and their preference for public transportation by understanding the significance of transportation options for energy conservation and environmental protection. In addition, the government should energetically popularize energy conservation and emission reduction technology, and emission reduction products.

## 6. Conclusions

In this study, from the perspective of carbon density, the “two layer complete decomposition method” and LMDI method are employed to investigate the driving factors of carbon emissions generated by the final energy consumption in the case of Shandong Province in China. The main findings are presented as follows.

(1) Overall, from 2000 to 2015 in Shandong Province, carbon density increased, following an annual growth rate of 7.21%. The positive driving factors comprised eight factors including per capita GDP, traffic congestion, land urbanization, etc. The negative driving factors were seven factors, including energy mix, economic structure, energy intensity of production, etc. Per capita GDP was the main positive driving factor, and it was always the leading driving factor causing increases in carbon density. As industrialization and urbanization progressed, the traffic congestion and land urbanization gradually became the second and third largest positive driving factors, respectively. The energy intensity of production was the primary negative driving factor. Thus, technological progress was the key measure for emission reduction. The inhibiting effect of economic structure on carbon density gradually increased. However, the inhibiting effect of energy mix was not obvious, and the potential was not released.

(2) In the classification of factors influencing carbon density, the economic effect was the leading positive driving force, and the accumulative contribution was 164.93%. The energy intensity effect was the leading restraint on carbon density, following an accumulative contribution rate of −84.36%. The accumulative contribution rates of the structural effect and scale effect on carbon density were −15.76% and −10.68%, respectively. Based on the population, land urbanization, and traffic congestion, there was positive driving effect of urbanization on carbon density, and the accumulative contribution was 45.87%.

## Figures and Tables

**Table 1:** Definition of variables in this study.

Variable	Definition	Variable	Definition
Carbon density (CD)	Carbon emissions/Administrative area	Average urban income (AUI)	Total urban income/urban population
Carbon emission coefficient (CEC)	Carbon emissions per unit *j*-th energy consumption	TUI	Total urban income
$C^{ij}$	Carbon emissions from the *j*-th energy consumption produced by the *i*-th sector	TRI	Total rural income
$F^{ij}$	The *j*-th energy consumption by the *i*-the sector	Average rural income (ARI)	Total rural income/rural population
$F^{i}$	Total energy consumption by the *i*-th sector	PCG	Per capita gross domestic production
$Y^{i}$	Output by the *i*-th sector	$CI^{ij}$	Carbon intensity of the *j*-th energy in the *i*-th sector
Y	Gross output	$FS^{ij}$	Proportion of the *j*-th energy consumption in the *i*-th sector
P	Total population	$EIP^{i}$	Energy intensity of the *i*-th sector
UP	Urban population	$ES^{i}$	Ratio of output of the *i*-th sector to total output
RP	Rural population	EIT	Energy intensity of transportation
S	Administrative area	Energy intensity of production (EIP)	Total energy consumption by the *i*-th sector ($F^{i}$)/Output by the *i*-th sector ($Y^{i}$)
Population density (PD)	Total population/administrative area	EIU	Energy intensity of urban residents
Urban population density (UPD)	Urban population/administrative area	EIR	Energy intensity of rural residents
Rural population density (RDP)	Rural population/administrative area	$S_{load}$	Road area
TD	Transport distance	Traffic congestion (TC)	Road area/vehicles number
VN	Vehicles number	Land urbanization (LU)	Road area/administrative area
Average transport distance (ATD)	Transport distance/ vehicles number		

**Table 2:** Classification of driving factors.

Effect	Factor
Energy intensity effect	Energy intensity of production (EIP)
	Energy intensity of transportation (EIT)
	Energy intensity of urban residents (EIU)
	Energy intensity of rural residents (EIR)
Structural effect	Energy mix (EM)
	Economic structure (ES)
Economic Effect	Per capita GDP (PCG)
	Average urban income (AUI)
	Average rural income (ARI)
Scale effect	Population density (PD)
	Average transport distance (ATD)
Urbanization effect	Traffic congestion (TC)
	Land urbanization (LU)
	Urban population density (UPD)
	Rural population density (RPD)

## Appendix A. Derivation of Related Carbon Density Decomposition

The derivative of both sides of Equation (2) are taken with respect to time, and then the integral is computed in the range of 0–T, yielding Equation (A1).

(A1) $$\begin{array}{ll} CD^{T} & -CD^{0}=\int_{0}^{T}\frac{dCD}{dt}dt=\int_{0}^{T}(\sum_{i=1}^{4}\sum_{j=1}^{3}\frac{C^{ij}}{CEC^{ij}}•\frac{dCEC^{ij}}{dt}+\frac{C^{ij}}{EM^{ij}}•\frac{dEM^{ij}}{dt}+\frac{C^{ij}}{EIP^{i}}•\frac{dEIP^{i}}{dt}\\ & +\frac{C^{ij}}{ES^{i}}•\frac{dES^{i}}{dt}+\frac{C^{ij}}{PCG}•\frac{dPCG}{dt}+\frac{C^{ij}}{PD}•\frac{dPD}{dt})dt\\ & +\int_{0}^{T}(\sum_{i=5}^{}\sum_{j=1}^{3}\frac{C^{ij}}{CEC^{ij}}•\frac{dCEC^{ij}}{dt}+\frac{C^{ij}}{EM^{ij}}•\frac{dEM^{ij}}{dt}+\frac{C^{ij}}{ETI}•\frac{dETI}{dt}+\frac{C^{ij}}{ATD}•\frac{dATD}{dt}\\ & +\frac{C^{ij}}{TC^{-1}}•\frac{d{\left(TC\right)}^{-1}}{dt}+\frac{C^{ij}}{LU}•\frac{dLU}{dt})dt+\int_{0}^{T}(\sum_{i=6}^{}\sum_{j=1}^{3}\frac{C^{ij}}{CEC^{ij}}•\frac{dCEC^{ij}}{dt}+\frac{C^{ij}}{EM^{ij}}•\frac{dEM^{ij}}{dt}\\ & +\frac{C^{ij}}{EIU}•\frac{dEIU}{dt}+\frac{C^{ij}}{AUI}•\frac{dAUI}{dt}+\frac{C^{ij}}{UPD}•\frac{dUPD}{dt})dt\\ & +\int_{0}^{T}(\sum_{i=7}^{}\sum_{j=1}^{3}\frac{C^{ij}}{CEC^{ij}}•\frac{dCEC^{ij}}{dt}+\frac{C^{ij}}{EM^{ij}}•\frac{dEM^{ij}}{dt}+\frac{C^{ij}}{EIR}•\frac{dEIR}{dt}\\ & +\frac{C^{ij}}{ARI}•\frac{dARI}{dt}+\frac{C^{ij}}{RPD}•\frac{dRPD}{dt})dt\\ & =\int_{0}^{T}\sum_{i=1}^{4}\sum_{j=1}^{3}C^{ij}(\frac{d\ln CEC^{ij}}{dt}+\frac{d\ln EM^{ij}}{dt}+\frac{d\ln EIP^{i}}{dt}+\frac{d\ln ES^{i}}{dt}+\frac{d\ln PCG}{dt}+\frac{d\ln PD}{dt})dt\\ & +\int_{0}^{T}\sum_{i=5}^{}\sum_{j=1}^{3}C^{ij}(\frac{d\ln CEC^{ij}}{dt}+\frac{d\ln EM^{ij}}{dt}+\frac{d\ln EIT}{dt}+\frac{d\ln ATD}{dt}-\frac{d\ln TC}{dt}+\frac{d\ln LU}{dt})dt\\ & +\int_{0}^{T}\sum_{i=6}^{}\sum_{j=1}^{3}C^{ij}(\frac{d\ln CEC^{ij}}{dt}+\frac{d\ln EM^{ij}}{dt}+\frac{d\ln EIU}{dt}+\frac{d\ln AUI}{dt}+\frac{d\ln UPD}{dt})dt\\ & +\int_{0}^{T}\sum_{i=7}^{}\sum_{j=1}^{3}C^{ij}(\frac{d\ln CEC^{ij}}{dt}+\frac{d\ln EM^{ij}}{dt}+\frac{d\ln EIR}{dt}+\frac{d\ln ARI}{dt}+\frac{d\ln RPD}{dt})dt \end{array}$$

Detailed derivation process can refer to Ang and Wang [67]. $X_{k}$ represents the *k*-th driving factor of carbon density.

(A2) $$CD^{T}-CD^{0}=\sum_{i}^{}\sum_{j}\left(\omega_{ij}(t^{*})\ln\frac{X_{1}^{ijT}}{X_{1}^{ij0}}+\omega_{ij}(t^{*})\ln\frac{X_{2}^{ijT}}{X_{2}^{ij0}}+\cdots +\omega_{ij}(t^{*})\ln\frac{X_{16}^{ijT}}{X_{16}^{ij0}}\right)\;$$

In the above formula, traffic congestion is different from other variables due to the negative sign in front of it. Then, we can assume that $X_{TC}$ represents traffic congestion. Equation (A2) can be also expressed as Equation (A3)

(A3) $$CD^{T}-CD^{0}=\sum_{i}^{}\sum_{j}\left(\omega_{ij}(t^{*})\ln\frac{X_{1}^{ijT}}{X_{1}^{ij0}}+\omega_{ij}(t^{*})\ln\frac{X_{2}^{ijT}}{X_{2}^{ij0}}+\cdots +\omega_{ij}(t^{*})\ln\frac{X_{15}^{ijT}}{X_{15}^{ij0}}+\omega_{ij}(t^{*})\ln\frac{X_{TC}^{ij0}}{X_{TC}^{ijT}}\right)\;$$
